# Sparse trees and shrubs confers a high biodiversity to pastures: Case study on spiders from Transylvania

**DOI:** 10.1371/journal.pone.0183465

**Published:** 2017-09-08

**Authors:** Róbert Gallé, István Urák, Gallé-Szpisjak Nikolett, Tibor Hartel

**Affiliations:** 1 Department of Ecology, University of Szeged, Szeged, Hungary; 2 Ecosystem Services Laboratory, Department of Environmental Sciences—Sapientia Hungarian University of Transylvania, Cluj-Napoca, Romania; 3 Department of Taxonomy and Ecology, Faculty of Biology, Babes-Bolyai University, Cluj-Napoca, Romania; Charles University, CZECH REPUBLIC

## Abstract

The integration of food production and biodiversity conservation represents a key challenge for sustainability. Several studies suggest that even small structural elements in the landscape can make a substantial contribution to the overall biodiversity value of the agricultural landscapes. Pastures can have high biodiversity potential. However, their intensive and monofunctional use typically erodes its natural capital, including biodiversity. Here we address the ecological value of fine scale structural elements represented by sparsely scattered trees and shrubs for the spider communities in a moderately intensively grazed pasture in Transylvania, Eastern Europe. The pasture was grazed with sheep, cattle and buffalo (*ca* 1 Livestock Unit ha^-1^) and no chemical fertilizers were applied. Sampling sites covered the open pasture as well as the existing fine-scale heterogeneity created by scattered trees and shrub. 40 sampling locations each being represented by three 1 m^2^ quadrats were situated in a stratified design while assuring spatial independency of sampling locations. We identified 140 species of spiders, out of which 18 were red listed and four were new for the Romanian fauna. Spider species assemblages of open pasture, scattered trees, trees and shrubs and the forest edge were statistically distinct. Our study shows that sparsely scattered mature woody vegetation and shrubs substantially increases the ecological value of managed pastures. The structural complexity provided by scattered trees and shrubs makes possible the co-occurrence of high spider diversity with a moderately high intensity grazing possible in this wood-pasture. Our results are in line with recent empirical research showing that sparse trees and shrubs increases the biodiversity potential of pastures managed for commodity production.

## Introduction

The integration of commodity production and biodiversity conservation in agricultural landscapes is an increasing challenge for sustainability [1]. Traditional farming landscapes are especially important in meeting this challenge because these landscapes have exceptional ecological values and evolved as commodity (food, timber, vine) production landscapes [2], [3], [4]. Within this context, traditionally managed pastures with small natural features such as the sparse trees and shrubs are considered archetypes of traditional landscapes with high natural and cultural values in Europe [5]. Sparse mature trees and shrubs provided several benefits for the local farmer communities including shadow for livestock, fruits, halting soil erosion and cultural-aesthetic values (overviewed e.g. in [6]). Besides their important socio-economic roles, scattered trees are considered keystone habitat structures [7], [8]. Solitary trees on pastures significantly increased the overall diversity of lichens [9], vascular plants and bryophytes [10]. Sparse trees are important breeding sites for birds [11], [12] and they may also harbor a great invertebrate diversity [13], [14]. Trees create a distinct microclimatic conditions on grasslands and this results in distinct arthropod species assemblages, including several rare species [15], [16], [17]. Despite their several ecological and socioeconomic values, the sparse woody vegetation from pastures is seriously threatened because the current formal institutions (e.g. the Common Agricultural Policy (CAP) and the national level institutions) does not explicitly recognize their multiple values and potentials [16]. CAP negatively affects sparse trees on pastures mainly through the lack of explicit recognition of their socio-economic values, lack of incentives to farmers to regenerate them and their removal when these trees are perceived as ‘damaged’ (e.g. hollowing) [18]. Without an explicit policy recognition and support, sparsely scattered trees are going to disappear from pastures [5]. The removal of sparse trees from pastures will have substantial impact on the overall biodiversity, adaptive potential and ecological resilience of these commodity production landscapes [6]. The main goal of this contribution is to document the ecological importance of sparse trees and shrubs for the overall pasture biodiversity in a moderately intensively grazed pasture from (Transylvania, Romania).

Spiders are abundant invertebrate predators in several terrestrial ecosystems and their taxonomy together with their life histories are relatively well-known [19]. Spiders respond to structural changes in vegetation, soil moisture and shade even in very small spatial scales [20], [21], [22]. The importance of fine-scale habitat heterogeneity created by woody vegetation for spider species assemblages was explored by several studies in Europe. These studies targeted a wide range of ecosystems including riparian areas e.g. [23], [24], dry steppe woodlands e.g. [25], subalpine forests e.g. [26], [27], mature forests e.g. [28], coppice forests e.g. [29], retention trees on clear-cuts e.g. [30] or the Mediterranean regions e.g. [31]. To the best of our knowledge, traditional pastoral systems such are those from Eastern Europe are still not explored regarding the importance of sparse trees on pastures to spider communities. In order to fill this knowledge gap, we selected a lowland area within a highly productive region of Transylvania (Romania), which was managed by grazing since historical times (see below). Agricultural production is typically intensified in such landscapes when modern technology becomes available and as a result, sparse trees are likely to be removed [32]. The aim of the present study was to assess the importance of scattered trees in an open grazed landscape for spider communities. More specifically, we hypothesized that sparsely scattered trees and shrubs creates distinct spider species assemblages compared to the pasture surfaces without these structural elements and thus confers a high beta diversity value for the grazed system.

## Materials and methods

### Study area and methods

#### The description of the pasture and its management

**Fig 1:**
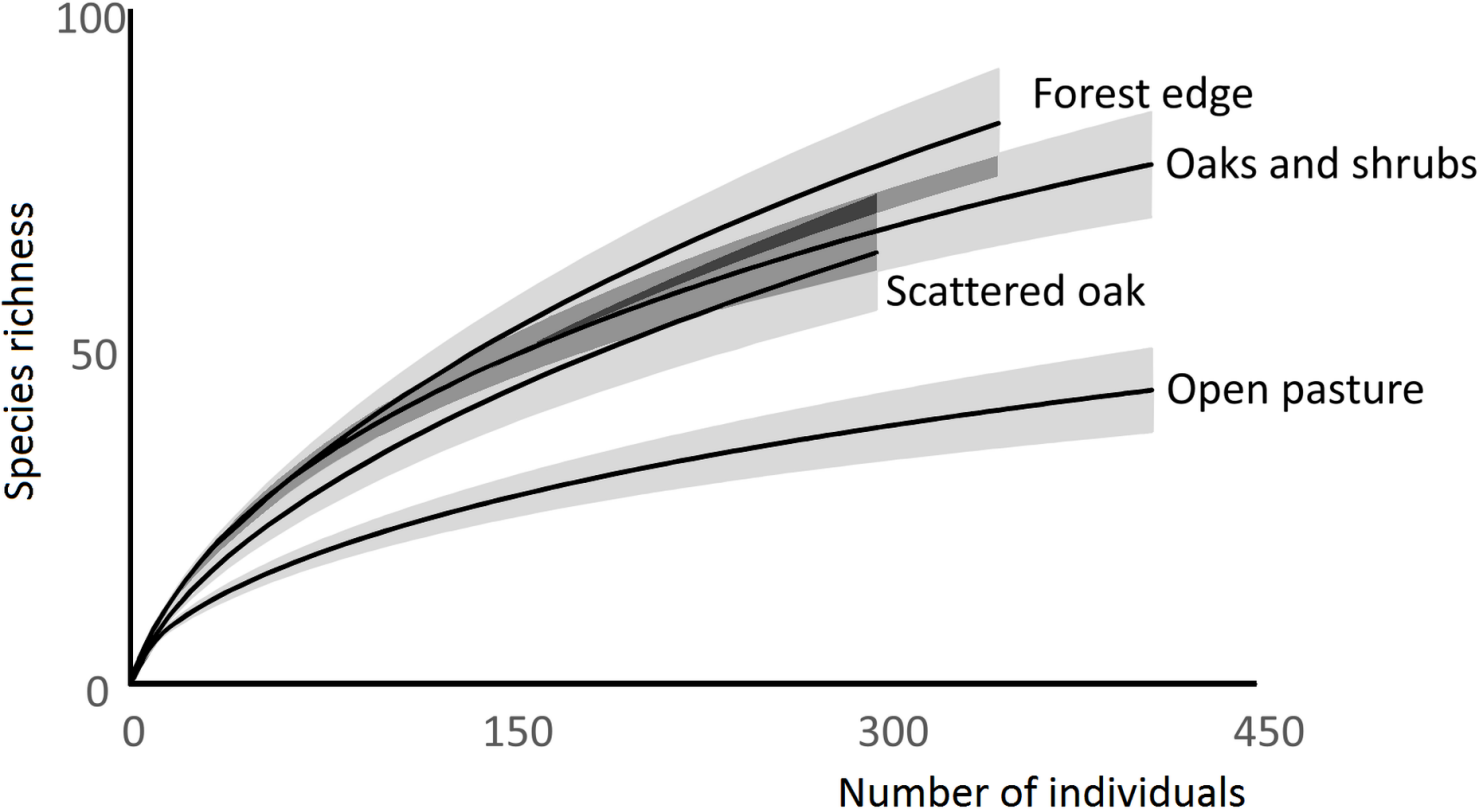
Species accumulation curves for the four habitat types of the studies wood-pasture. Gray shades represent 95% confidence intervals.

The studied pasture was situated in the Transylvanian region of Romania at an altitude of *ca* 430 m (central coordinates: Lat: 46.9198°; Long: 23.5053°). The surface area of the pasture was *ca* 250 hectare (see Fig 1 for map). The study site was situated within the continental biogeographic region. The temporal continuity of livestock grazing in the study area covered at least two centuries. This area was visualized as semi-open landscape in the 18^th^ century Josephine maps and historical descriptions of woodland management in Transylvania e.g. [33] suggests that these landscapes were grazed either with pig or by cattle.

The current physiognomy of the pasture is presented in S1 Fig. The pasture contains sparse trees, in an overall density of *ca* 1 tree per hectare. This physiognomy emerged likely in the end of the 19^th^–early 20^th^ century, when several woodlands were opened and transformed in open pastures while sparsely scattered trees were maintained for shadow and/or fruits [33]. At the eastern part, the pasture is bordered by a high oak forest managed for timber production where grazing is prohibited. The sparse trees on the studied pasture were represented mainly by mature oak (*Quercus robur* and *Q*. *cerris* in equal proportion) while shrubs were represented by *Prunus*. sp, *Rosa*. sp and *Crataegus* sp. During the period of our study the pasture was grazed by sheep, cattle and buffalo with an overall *ca* 0.93–1.1 livestock unit per hectare (local shepherds, personal communication), this grazing management being applied for at least four consecutive years. No chemical fertilizers were applied to this pasture at least for the past 50 years (shepherds and locals, personal communication). While the cattle and buffalo were allowed on the pasture typically in the period of May-November, the sheep grazing occurs through the whole year (local shepherds, personal communication, and author’s personal observation).

#### Sampling design and procedure

In order to assess the ecological importance of sparsely scattered trees on the grazed pasture, we placed 40 sampling sites were across the pasture, each site consisting from three square meter plots situated in a triangle, with 4–5 m distance between them. In the case of forest edge (see below) the three plots were situated in a linear way along the edge of the forest.

When selecting the sites we considered the following aspects: (i) To capture the existing structural gradients represented by woody vegetation across the pasture. In this way we ended up in studying four habitat types, which are presented below. (ii) To assure an even distribution of the sampling sites across the studied pasture surface and (iii) To assure that the neighboring sampling locations are distant enough to consider them ‘independent’ sampling units (see below). The distance between the neighboring sampling sites was set to at least 110 m (mean ± SD = 149.8 ± 33.4 m) in order to assure their spatial independence. We distributed the sampling sites across the pasture to cover the main structural (and grazing) gradients ranging from pasture surfaces without any tree and shrub to pasture surfaces with sparse trees and shrubs. These are detailed in the following. (i) The open pasture surfaces contained no woody vegetation. These sites were dominated by *Agrostis capillaris* and *Festuca stricta*. Based on the personal communication of shepherds, these pasture surfaces contained no woody vegetation for at least two decades and were continuously grazed. (ii) Scattered open grown mature oak trees. The tree sampling plots for this habitat were situated under the tree canopy positioned in a triangle around the trunk of the tree. The girth of the trees in the sampling sites was ranging between 2 and 3 m trunk circumference measured at *cca* 130 cm height (iii) Sparse trees with shrubs under their canopy, with the dominant shrubs *Prunus* sp., *Rosa* sp. and *Crataegus* sp. We considered ‘shrubs’ as present when we found a substantial coverage of shrub of at least 50 cm height under the tree canopy and/or in the very close vicinity (i.e. at least up to *cca* 2–3 m distance from the crown) of the tree. Shrubs typically develop in pastures from this region as a result of the decreasing grazing intensity. Indeed, the grazing intensity in Transylvania decreased with the overall abandonment of the farming landscape management in the decade following the collapse of the communism (i.e. 1989) [34] (iv) Forest edges with the herbaceous vegetation characterized by several forest and edge specialist plant species. Since the forest edges was regularly used as shading place by sheep, the forest-pasture transition (i.e. the ‘ecotone’) was abrupt, shrubs being only scarcely developed along the forest edge. In the following we refer to the above four structural elements as ‘habitat types’. Ten sites were selected in each of the four habitat types. The distance to the closest forest did not differ significantly between open pasture, scattered oak, tree and shrub sites (ANOVA, F = 1.298, p = 0.29), hence we assume that the variation in the spider communities detected in our study did was not related to the distance of the site from the forest. No autocorrelation of the spider fauna was detected by the mantel test between the Bray-Curtis dissimilarity matrix of spiders and the Euclidian distance between sampling sites (Mantel z = 4.693, p = 0.473).

The description of the habitat types was performed on 7–9 June in 2015 before the start of the spider sampling. We described each habitat type with four structural variables. Each of these variables were quantified in the 1m^2^ square plots which formed a sampling site (see above). These variables were: (i) *Average herbaceous vegetation height*: the height of the vegetation in cm, measured in three random points; the average value of these measurements were used for each site (ii) *Herbaceous vegetation cover*: the average percentage of vegetation cover. (iii) *Liter coverage*: the leaf litter coverage, estimated visually by three persons. (iv) *Bare ground coverage*: the percentage of bare ground, estimated visually by three persons and averaged. The variables characterizing the sampling sites were presented using descriptive statistics: average, 95% confidence intervals (Table 1).

**Table 1:** Descriptive characteristics of the vegetation cover of the habitat types.

Habitat type/Habitat features	Average herbaceous vegetation height (cm)	Herbaceous vegetation cover (%)	Bare ground cover (%)	Litter cover (%)
Open pasture	10.2 (7.8, 12.6)	99.4 (98.6, 100)	0.1 (0, 0.2)	0
Scattered oak	10.9 (8.8, 13.1)	90.8 (84.7, 96.9)	4.2 (2.1, 6.3)	5.2 (2.6, 1.3)
Scattered trees with shrubs	14.2 (12.4, 16.1)	85.5(79.9, 91.0)	4.2 (1.1, 7.2)	17.3 (12.1, 22.5)
Forest edge	11.1 (9.4, 12.8)	63.8 (56.5, 71.1)	12.0 (7.2, 16.8)	31.2 (19.4, 42.9)

Mean value; standard deviation and coefficient of variation are given in parenthesis.

Spiders were collected in middle of May (two consecutive days: 12 and 13) and early June (three consecutive days (07–09) in 2015 using a ‘D-vac’ suction sampler. Spiders were collected only from the herbaceous vegetation; no sampling occurred from the canopy of shrubs and trees. The ‘D-vac’ head was placed near the ground in order to increase efficiency in sampling the ground dwelling spiders. Spiders caught with suction sampling were placed in 70% alcohol-water solution and adult specimens were identified to species level in the laboratory.

### Data analysis

The effect of habitat type on species richness of spider species of conservation interest were tested with general linear model (GLM) with Poisson error term and log-link function. We tested the effect of habitat type on abundance of spider species of conservation interest with negative binomial GLM with logit link function, followed by the Tukey type pairwise comparisons using the glht function with p<0.05 from the multcomp library [35]. We computed species accumulation curves on the basis of pooled individual-based abundance data for open pasture, scattered oak, forest edge and tree and shrub sites to compare the species richness of spiders [iNEXT package, 36]. We also estimated the number of species and Shannon diversity for the pooled data of each habitat type with the ChaoRichness and ChaoShannon functions, iNEXT package [36].

The multivariate response of spider communities to habitat type was studied with Non-metric Multidimensional Scaling (NMDS) on the basis of Bray-Curtis dissimilarity matrix. We pooled the data from the two collection dates and the three samples per site for this analysis. A Hellinger transformation was applied to the data prior to the ordination [37]. The abundance of each trait was fitted passively onto the NMDS ordination plot and the correlation between the species traits and habitat types (see above) was tested by 5000 Monte-Carlo permutations [38] and traits with significant correlation (p<0.05) were displayed on NMDS plot. Analysis of Similarities (ANOSIM) with 5000 permutations was performed to test the difference among the four habitat types based on Bray-Curtis dissimilarity matrices. ANOSIM is a multivariate analysis of variance that uses dissimilarity between pairs of samples [39]. We considered each site as a replicate, we summed abundances of each species over three plots and two sampling periods. All habitats were compared by pairs to test whether there is a significant difference among them (ANOSIM, Bray-Curtis dissimilarity; 5000 permutations followed by a Bonferroni-Holm adjustment of P-values).

## Results

### Characterizing the four habitat types

The average height of the herbaceous vegetation across the four habitat types varied very narrowly between 10 and 12 cm (Table 1). The herbaceous vegetation cover was highest as expected in the open pasture habitat (99%) and lowest (64%) in the forest edge (Table 1). The coverage of bare ground was highest on the forest edges (12%) while in the other three habitat types was below 5% (Table 1). Finally, the litter cover was highest in the forest edges (31%) followed by tree and shrub habitat (17%) and scattered tree habitat (5%), while the open pasture habitat had no litter (Table 1).

### Spider fauna

We collected 2697 spider specimens (1461 adults), belonging to 140 species from 21 families (S1 Table). Two species of spiders were new for the Romanian fauna (*Synageles subcingulatus*, one specimen at scattered tree habitat and *Talavera parvistyla* one specimen at tree and shrub habitat). Two species (*Dipoena erythropus*, two individuals identified from open pasture and scattered tree sites habitats and *Philodromus praedatus*, two individuals from open pasture and forest edge habitats) with uncertain presence for the Romanian fauna were confirmed by the present study. We found 25 species of spiders which are considered rare (Table 2). These species are typically considered as preferring ‘climax’, ‘non-disturbed’ and ‘natural habitats’ in Europe e.g. [40], [41][42]. We found no significant effect of habitat type on species richness of rare species according to the Poisson GLM and the following pairwise comparisons. However sparse trees with shrubs had significantly higher abundance of rare species than scattered oaks (z = 3.213, p = 0.006).

**Table 2:** Abundances of species of conservation interest. Species and categories according to [41] and [42].

	Open pasture	Forest edge	Oaks and shrubs	Scattered oaks	sum
Endangered					
*Drassyllus pumilus* (C. L. Koch, 1839)	0	1	1	0	2
*Mecopisthes silus* (O. P.-Cambridge, 1872)	0	5	0	0	5
Vulnerable					
*Agyneta simplicitarsis* (Simon, 1884)	6	0	4	1	11
*Dipoena erythropus* (Simon, 1881)	1	0	0	1	2
*Metopobactrus ascitus* (Kulczyński, 1894)	0	0	1	0	1
*Micaria dives* (Lucas, 1846)	0	1	3	0	4
*Micaria formicaria* (Sundevall, 1831)	0	0	1	0	1
*Microdipoena jobi* (Kraus, 1967)	0	0	1	0	1
*Zelotes electus* (C. L. Koch, 1839)	0	0	0	1	1
Least concern					
*Anelosimus vittatus* (C. L. Koch, 1836)	0	0	0	2	2
*Aulonia albimana* (Walckenaer, 1805)	0	0	3*	0	3
*Carrhotus xanthogramma* (Latreille, 1819)	0	2	1	1	4
*Dysdera hungarica* Kulczyński, 1897	0	1	0	0	1
*Erigonoplus globipes* (L. Koch, 1872)	0	1	0	0	1
*Nematogmus sanguinolentus* (Walckenaer, 1841)	0	1	0	1	2
*Neon valentulus* Falconer, 1912	1	0	0	1	2
*Panamomops mengei* Simon, 1926	0	0	2	0	2
*Pardosa monticola* (Clerck, 1757)	2	0	0	0	2
*Phlegra fasciata* (Hahn, 1826)	1	0	0	0	1
*Poecilochroa variana* (C. L. Koch, 1839)	0	0	1	0	1
*Synageles subcingulatus* (Simon, 1878)	0	0	1	0	1
*Talavera aperta* (Miller, 1971)	0	2	8	1	11
*Thanatus arenarius* L. Koch, 1872	0	1	0	0	1
*Titanoeca quadriguttata* (Hahn, 1833)	0	1	0	0	1
*Trichoncus affinis* Kulczyński, 1894	0	2	8	0	10
*sum*	11	18	32	9	73

### Spider communities

Tree and shrub sites had the highest spider species richness (19.3, 95% confidence interval [CI]: 16.3–22.2) followed by forest edge (17.3, 95% CI, 13.8–20.7), scattered tree (14.5 95% CI, 12.1–16.8) and open pasture (12.8, 95% CI, 9.58–16.01) habitats. The number of spider species detected exclusively in the open pasture habitat was seven (representing 16% of the species found in this habitat type), while for the scattered tree habitat this value was eight (13%), for tree and shrub 25 (32%) and for forest edge 25 (30%). The estimated species richness Shannon diversity and species accumulation curves also detected lower diversity of open pasture sites compared to sampling sites with woody vegetation (Table 3, Fig 1).

**Table 3:** Estimated species richness and Shannon diversity of the four habitat types. Mean values and 95% confidence intervals are given.

	Estimated species richness	Estimated Shannon diversity
Open pasture	80.0 (55.0–161.3)	2.71 (2.614–2.86)
Scattered oak	156.2 (99.6–302.5)	3.49 (3.23–3.64)
Scattered trees with shrubs	111.0 (91.1–158.7)	3.73 (3.58–3.88)
Forest edge	140.6 (109.3–209.0)	3.75 (3.56–3.93)

**Fig 2:**
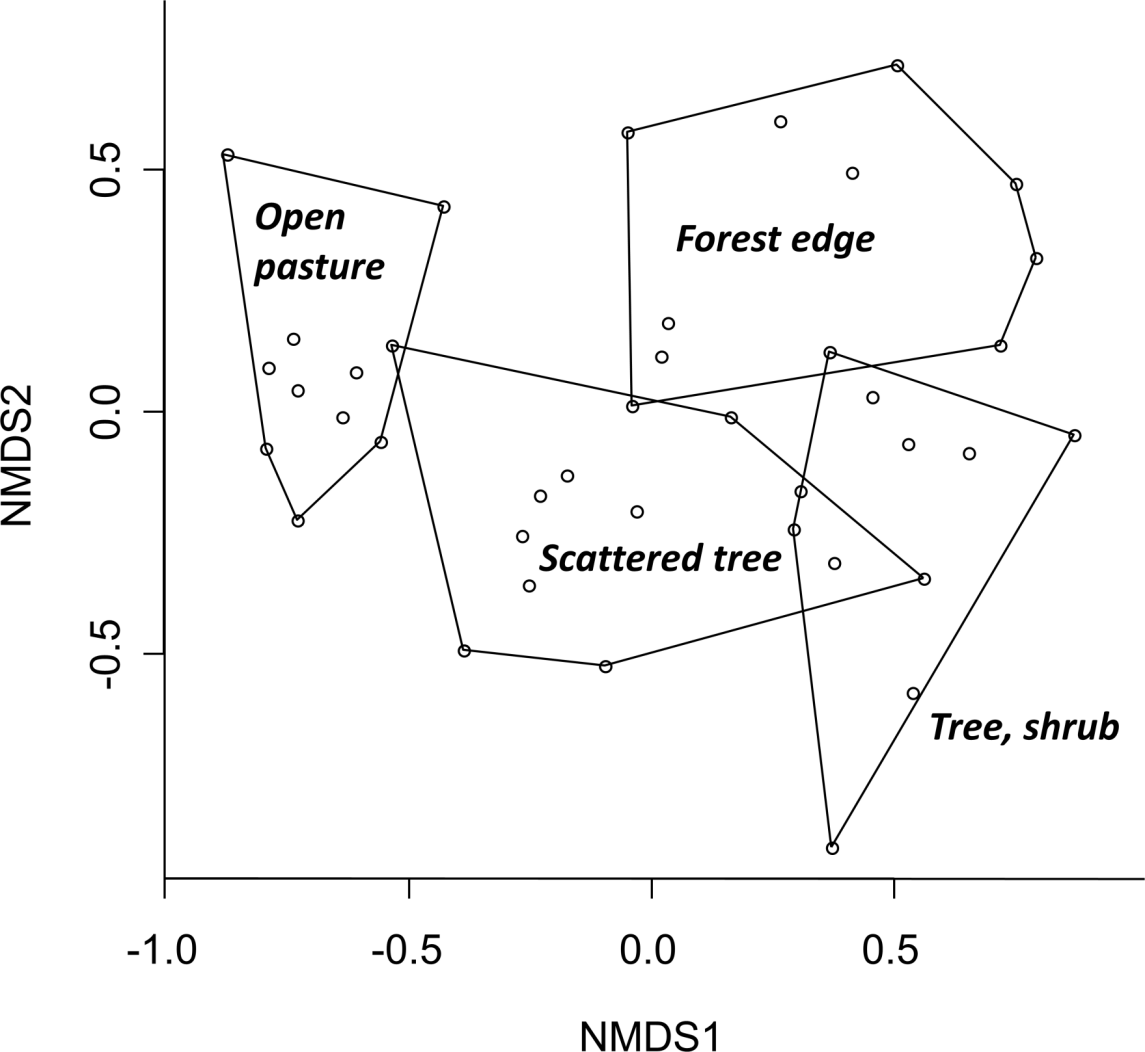
Non-metric multidimensional scaling of spider species assemblages of the four habitat types studied. Open circles represent the sampling sites. Stress: 0.201.

Ordination by NMDS indicated a clear separation of species composition among the habitat types with relatively small overlap between the polygons grouping the four habitat types (Fig 2). The ANOSIM statistic highlighted significant difference among the four habitat types (R = 0.611, P<0.001). Furthermore, the subsequent comparison of habitat types showed significant differences in spider community structure between all pairs of habitat types (ANOSIM, P_adjusted_<0.01).

## Discussion

We explored the importance of fine-scale heterogeneity for spider assemblages in a moderately intensively grazed pasture from Eastern-Europe). We highlight a remarkably high number of spider species in the studied pasture, which also includes four previously unknown species for Romania and several species which are considered rare in Europe. Fine-scale structural elements represented by sparse trees and shrubs had significant influence on the spider species assemblages from the ground and low vegetation.

Our results showed that small natural features such as the sparsely scattered trees and shrubs confers a high biodiversity value–as measured through spider communities—for managed pastures, this is in line with several studies investigating the effect of sparsely scattered trees on Arthropods (Table 4). Scattered trees with shrubs had higher spider species richness than the other three habitat types but each of the four habitat type contributed to the beta diversity of the studied pasture through statistically distinct spider community assemblages. The high potential of the scattered trees on pastures for spiders of conservation interest is also highlighted by the fact that we identified new species for the Romanian spider fauna under the canopy of these trees. Solitary trees had different spider species assemblages than the surrounding open landscape in the alpine timberline [42] while open-grown tree canopies harbored high number of spider species and high of conservation value assemblages in a lowland area from Czech Republic [15].

**Table 4:** Examples of studies investigating the effect of solitary large old trees and shrubs on different Arthropod taxa.

Arthropods	References
Pseudoscorpions	[43]
Collembola	[44], [45]
Coleoptera (e.g. saproxylic Cearmbicidae, Scarabaeidae, Carabidae)	[16], [17], [45], [46], [47], [48], [49], [50]
Diptera (e.g. Syrphidae, Culicidae)	[45], [49]
Hymenoptera (e.g. Formicidae, wasps and bees)	[17], [51]
Lepidoptera	[52], [53]
Araneae	[17], [27], [54]

Sparse trees and shrubs on pastures results in a substantial improvement of the ecological conditions of the pasture by creating habitat niches for several species of spiders. Several of these species may have high conservation status (see Results). This result was surprising and unexpected, since the studied pasture was relatively intensively managed by livestock grazing. In this respect, our results suggest that the pasture with sparse trees grazed by livestock may act as ‘wild’ landscape for several species of spiders.

The statistical differentiation of the spider species assemblages in the four habitat types was possible due to the ‘unique’ spider species which were associated to specific habitats (see Results).

Vegetation structure, shading and microclimate are considered major divers of spider community structure [22]. Scattered trees provide shade and moist and intercept direct radiation and precipitation [54], [55]. Significant litter accumulation under scattered trees provides habitat and food resources for several invertebrate decomposers which may act as prey items for spiders [56], [57]. Soil nutrient levels under oaks are enhanced by livestock excrement [7] while trampling by livestock represent disturbance; this results in distinct herbaceous plant and insect herbivore species communities which in turn also affects spider communities in these habitats (see our results).

Sparse woody vegetation is of crucial importance for the overall biodiversity of pastures [7], [8], [11], [12] (Table 4) and contributes to the soil fertility [14]. Still, this vegetation elements are not explicitly recognized by major policies and institutions responsible for the governance of the management of the pastoral systems [18]. Sparse trees are typically disfavored both by agricultural policies (either by the Common Agricultural Policy, and the national level agricultural policies) and the forestry policies. This situation resulted in the decline of treed pastures all over Europe [5], [58]. We showed that sparse woody vegetation can bring substantial contribution to the biodiversity of pastures. Hence it is of utmost importance to harmonize various policies relevant for the sustainability of these pasture ecosystems e.g. [5], [18], [59]. Pastures with sparse trees may well integrate food production and biodiversity conservation; our study suggest that the biodiversity and structural properties of these pastures are tightly linked.

## Supporting information

S1 Fig The map of the studied pasture with the four habitat types sampled. The physiognomy of the pasture and typical pictures representing the sampled habitats are also shown.
(TIF)

S1 Table List of collected species spider species and number of individuals collected in the wood-pasture with and without arboreal and shrub vegetation in Transylvania in May and June 2015. Significant indicator values (IndVal, Dufrêne & Legendre, 1997) are indicated with stars (p<0.05).
(DOCX)
