# Supplementary figures and images for: Sparse trees and shrubs confers a high biodiversity to pastures: Case study on spiders from Transylvania
Source: PLoS One. 2017 Sep 8;12(9):e0183465. doi: 10.1371/journal.pone.0183465 (PMC5590833; doi:10.1371/journal.pone.0183465)

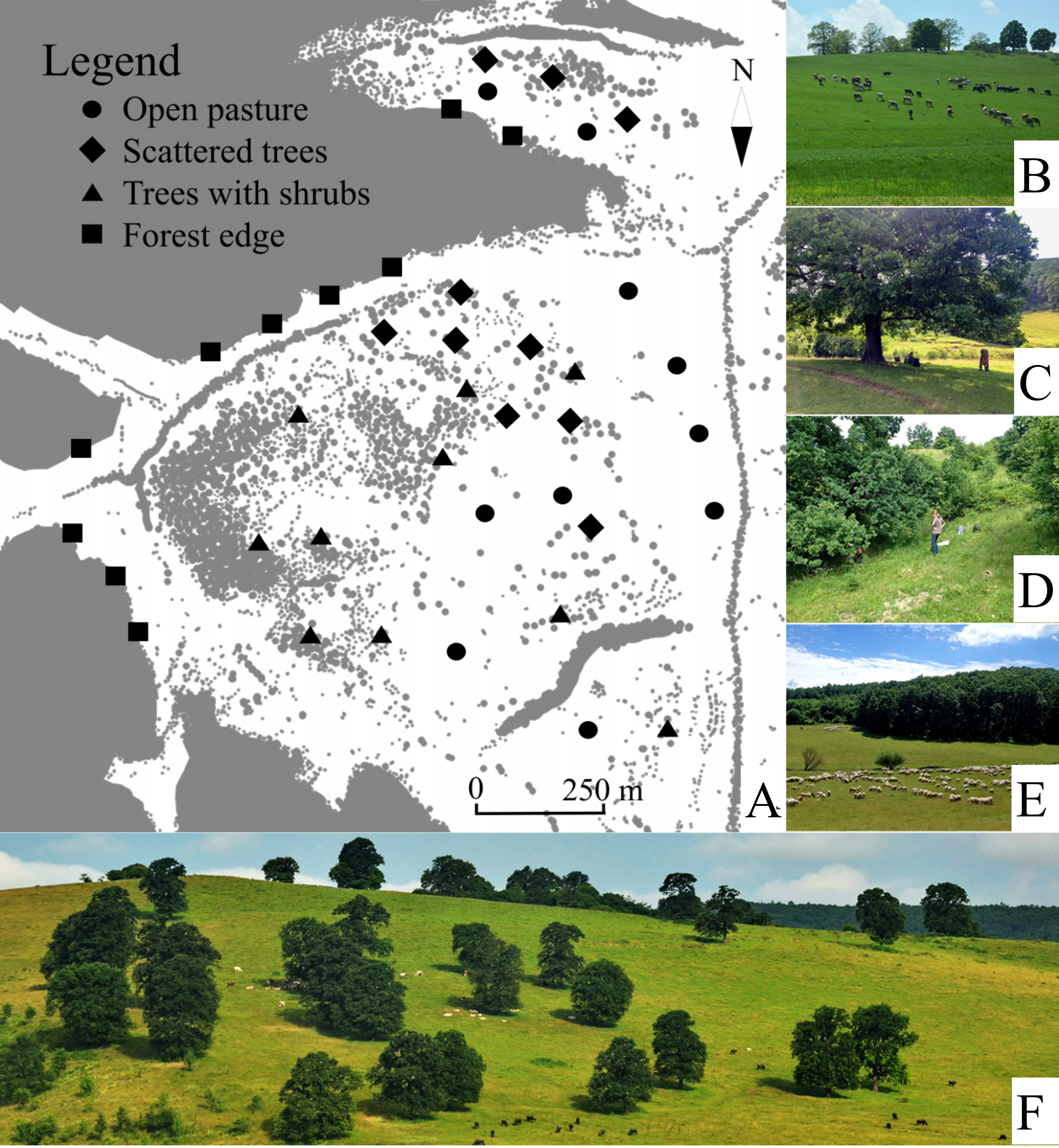

Supplement: S1 Fig — The physiognomy of the pasture and typical pictures representing the sampled habitats are also shown. (TIF) [file pone.0183465.s001.tif]
