# Supplementary material for: Sparse trees and shrubs confers a high biodiversity to pastures: Case study on spiders from Transylvania
Source: PLoS One. 2017 Sep 8;12(9):e0183465. doi: 10.1371/journal.pone.0183465 (PMC5590833; doi:10.1371/journal.pone.0183465)
Supplement: S1 Table — Significant indicator values (IndVal, Dufrêne & Legendre, 1997) are indicated with stars (p<0.05). (DOCX) [file pone.0183465.s002.docx]

**S1 Table. List of collected species Spider species and number of individuals collected in the wood-pasture with and without arboreal and shrub vegetation in Transylvania in May and June 2015.** Significant indicator values (IndVal, Dufrêne & Legendre, 1997) are indicated with stars (p<0.05).

|  | Open pasture | Forest edge | Oaks and shrubs | Scattered oaks | sum |
| --- | --- | --- | --- | --- | --- |
| *Abacoproeces saltuum* (L. Koch, 1872) | 0 | 5 | 16 | 0 | 21 |
| *Agroeca cuprea* Menge, 1873 | 0 | 1 | 0 | 0 | 1 |
| *Agyneta affinis* (Kulczyński, 1898) | 8 | 4 | 6 | 9 | 27 |
| *Agyneta mollis* (O. P.-Cambridge, 1871) | 47 | 31 | 30 | 49 | 157 |
| *Agyneta rurestris* (C. L. Koch, 1836) | 59 | 28 | 15 | 44 | 146 |
| *Agyneta simplicitarsis* (Simon, 1884) | 6 | 0 | 4 | 1 | 11 |
| *Alopecosa pulverulenta* (Clerck, 1757) | 3 | 0 | 2 | 3 | 8 |
| *Anelosimus vittatus* (C. L. Koch, 1836) | 0 | 0 | 0 | 2 | 2 |
| *Araeoncus humilis* (Blackwall, 1841) | 20* | 0 | 0 | 4 | 24 |
| *Araniella cucurbitina* (Clerck, 1757) | 1 | 2 | 3 | 4 | 10 |
| *Argenna subnigra* (O. P.-Cambridge, 1861) | 14* | 3 | 2 | 7 | 26 |
| *Asagena phalerata* (Panzer, 1801) | 2 | 1 | 0 | 0 | 3 |
| *Aulonia albimana* (Walckenaer, 1805) | 0 | 0 | 3* | 0 | 3 |
| *Ballus chalybeius* (Walckenaer, 1802) | 0 | 1 | 0 | 0 | 1 |
| *Bathyphantes gracilis* (Blackwall, 1841) | 0 | 1 | 0 | 2 | 3 |
| *Callilepis schuszteri* (Herman, 1879) | 0 | 1 | 0 | 0 | 1 |
| *Carrhotus xanthogramma* (Latreille, 1819) | 0 | 2 | 1 | 1 | 4 |
| *Centromerus sylvaticus* (Blackwall, 1841) | 0 | 0 | 1 | 0 | 1 |
| *Ceratinella brevis* (Wider, 1834) | 0 | 0 | 2 | 1 | 3 |
| *Cercidia prominens* (Westring, 1851) | 0 | 0 | 1 | 0 | 1 |
| *Civizelotes gracilis* (Canestrini, 1868) | 3 | 4 | 2 | 1 | 10 |
| *Clubiona comta* C. L. Koch, 1839 | 0 | 0 | 1 | 0 | 1 |
| *Clubiona diversa* O. P.-Cambridge, 1862 | 6 | 0 | 3 | 1 | 10 |
| *Crustulina guttata* (Wider, 1834) | 0 | 0 | 8* | 0 | 8 |
| *Dictyna uncinata* Thorell, 1856 | 0 | 1 | 0 | 0 | 1 |
| *Dicymbium nigrum* (Blackwall, 1834) | 0 | 1 | 0 | 0 | 1 |
| *Diplocephalus picinus* (Blackwall, 1841) | 0 | 0 | 0 | 1 | 1 |
| *Diplostyla concolor* (Wider, 1834) | 1 | 2 | 0 | 0 | 3 |
| *Dipoena erythropus* (Simon, 1881) | 1 | 0 | 0 | 1 | 2 |
| *Drassodes pubescens* (Thorell, 1856) | 0 | 0 | 1 | 0 | 1 |
| *Drassyllus praeficus* (L. Koch, 1866) | 0 | 0 | 1 | 0 | 1 |
| *Drassyllus pumilus* (C. L. Koch, 1839) | 0 | 1 | 1 | 0 | 2 |
| *Drassyllus pusillus* (C. L. Koch, 1833) | 1 | 0 | 1 | 0 | 2 |
| *Drassyllus villicus* (Thorell, 1875) | 0 | 2 | 0 | 0 | 2 |
| *Dysdera hungarica* Kulczyński, 1897 | 0 | 1 | 0 | 0 | 1 |
| *Enoplognatha ovata* (Clerck, 1757) | 0 | 2 | 0 | 1 | 3 |
| *Enoplognatha thoracica* (Hahn, 1833) | 2 | 1 | 3 | 4 | 10 |
| *Entelecara acuminata* (Wider, 1834) | 0 | 2 | 1 | 5 | 8 |
| *Episinus truncatus* Latreille, 1809 | 0 | 1 | 2 | 0 | 3 |
| *Erigone dentipalpis* (Wider, 1834) | 95* | 3 | 1 | 15 | 114 |
| *Erigonoplus globipes* (L. Koch, 1872) | 0 | 1 | 0 | 0 | 1 |
| *Ero aphana* (Walckenaer, 1802) | 0 | 0 | 1 | 0 | 1 |
| *Ero furcata* (Villers, 1789) | 0 | 0 | 0 | 1 | 1 |
| *Euophrys frontal*is (Walckenaer, 1802) | 3 | 2 | 8 | 2 | 15 |
| *Euryopis flavomaculata* (C. L. Koch, 1836) | 0 | 0 | 1 | 0 | 1 |
| *Evarcha arcuata* (Clerck, 1757) | 0 | 0 | 1 | 0 | 1 |
| *Gibbaranea bituberculata* (Walckenaer, 1802) | 0 | 0 | 3 | 0 | 3 |
| *Gnathonarium dentatum* (Wider, 1834) | 0 | 1 | 0 | 0 | 1 |
| *Hahnia nava* (Blackwall, 1841) | 3 | 1 | 0 | 1 | 5 |
| *Haplodrassus signifer* (C. L. Koch, 1839) | 0 | 2 | 0 | 0 | 2 |
| *Haplodrassus silvestris* (Blackwall, 1833) | 0 | 1 | 2 | 0 | 3 |
| *Harpactea rubicunda* (C. L. Koch, 1838) | 0 | 1 | 0 | 0 | 1 |
| *Heliophanus cupreus* (Walckenaer, 1802) | 1 | 16 | 26* | 4 | 47 |
| *Heliophanus flavipes* (Hahn, 1832) | 9* | 2 | 2 | 2 | 15 |
| *Heterotheridion nigrovariegatum* (Simon, 1873) | 0 | 3 | 2 | 0 | 5 |
| *Hypsosinga albovittata* (Westring, 1851) | 1 | 0 | 0 | 0 | 1 |
| *Hypsosinga heri* (Hahn, 1831) | 0 | 0 | 1 | 0 | 1 |
| *Hypsosinga pygmaea* (Sundevall, 1831) | 1 | 0 | 0 | 0 | 1 |
| *Hypsosinga sanguinea* (C. L. Koch, 1844) | 0 | 0 | 1 | 0 | 1 |
| *Lathys humilis* (Blackwall, 1855) | 0 | 0 | 2 | 1 | 3 |
| *Linyphia hortensis* Sundevall, 1830 | 0 | 3 | 0 | 0 | 3 |
| *Macaroeris nidicolens* (Walckenaer, 1802) | 0 | 0 | 0 | 2 | 2 |
| *Mangora acalypha* (Walckenaer, 1802) | 3 | 12 | 9 | 2 | 26 |
| *Maso sundevalli* (Westring, 1851) | 0 | 1 | 2 | 0 | 3 |
| *Mecopisthes silus* (O. P.-Cambridge, 1872) | 0 | 5 | 0 | 0 | 5 |
| *Metopobactrus ascitus* (Kulczyński, 1894) | 0 | 0 | 1 | 0 | 1 |
| *Micaria albovittata* (Lucas, 1846) | 0 | 1 | 0 | 0 | 1 |
| *Micaria dives* (Lucas, 1846) | 0 | 1 | 3 | 0 | 4 |
| *Micaria formicaria* (Sundevall, 1831) | 0 | 0 | 1 | 0 | 1 |
| *Micaria pulicaria* (Sundevall, 1831) | 0 | 1 | 0 | 1 | 2 |
| *Micrargus subaequalis* (Westring, 1851) | 3 | 2 | 1 | 4 | 10 |
| *Microdipoena jobi* (Kraus, 1967) | 0 | 0 | 1 | 0 | 1 |
| *Microlinyphia pusilla* (Sundevall, 1830) | 1 | 0 | 0 | 0 | 1 |
| *Microneta viaria* (Blackwall, 1841) | 0 | 1 | 0 | 0 | 1 |
| *Minicia marginella* (Wider, 1834) | 0 | 6 | 47* | 1 | 54 |
| *Misumena vatia* (Clerck, 1757) | 0 | 0 | 0 | 1 | 1 |
| *Nematogmus sanguinolentus* (Walckenaer, 1841) | 0 | 1 | 0 | 1 | 2 |
| *Neon reticulatus* (Blackwall, 1853) | 1 | 0 | 0 | 1 | 2 |
| *Neon valentulus* Falconer, 1912 | 1 | 0 | 0 | 1 | 2 |
| *Neottiura bimaculata* (Linnaeus, 1767) | 0 | 6 | 6 | 1 | 13 |
| *Neottiura suaveolens* (Simon, 1879) | 4 | 0 | 5 | 0 | 9 |
| *Neriene clathrata* (Sundevall, 1830) | 0 | 5 | 6 | 3 | 14 |
| *Ozyptila simplex* (O. P.-Cambridge, 1862) | 3 | 1 | 0 | 0 | 4 |
| *Pachygnatha degeeri* Sundevall, 1830 | 2 | 1 | 3 | 15* | 21 |
| *Panamomops mengei* Simon, 1926 | 0 | 0 | 2 | 0 | 2 |
| *Pardosa agrestis* (Westring, 1861) | 2 | 0 | 0 | 0 | 2 |
| *Pardosa alacris* (C. L. Koch, 1833) | 1 | 46* | 14 | 3 | 64 |
| *Pardosa cribrata* Simon, 1876 | 0 | 1 | 0 | 0 | 1 |
| *Pardosa hortensis* (Thorell, 1872) | 0 | 8* | 0 | 1 | 9 |
| *Pardosa lugubris* (Walckenaer, 1802) | 0 | 1 | 0 | 1 | 2 |
| *Pardosa monticola* (Clerck, 1757) | 2 | 0 | 0 | 0 | 2 |
| *Pardosa paludicola* (Clerck, 1757) | 0 | 0 | 1 | 1 | 2 |
| *Pardosa palustris* (Linnaeus, 1758) | 18* | 0 | 0 | 3 | 21 |
| *Pardosa riparia* (C. L. Koch, 1833) | 0 | 0 | 5 | 0 | 5 |
| *Pelecopsis radicicola* (L. Koch, 1872) | 0 | 0 | 1 | 1 | 2 |
| *Philodromus praedatus* O. P.-Cambridge, 1871 | 1 | 1 | 0 | 0 | 2 |
| *Phlegra fasciata* (Hahn, 1826) | 1 | 0 | 0 | 0 | 1 |
| *Pholcomma gibbum* (Westring, 1851) | 0 | 0 | 1 | 0 | 1 |
| *Phrurolithus festivus* (C. L. Koch, 1835) | 0 | 6 | 22* | 11 | 39 |
| *Phrurolithus minimus* C. L. Koch, 1839 | 0 | 1 | 1 | 0 | 2 |
| *Phylloneta impressa* (L. Koch, 1881) | 5 | 0 | 1 | 3 | 9 |
| *Pisaura mirabilis* (Clerck, 1757) | 0 | 1 | 0 | 2 | 3 |
| *Platnickina tincta* (Walckenaer, 1802) | 0 | 0 | 1 | 1 | 2 |
| *Pocadicnemis pumila* (Blackwall, 1841) | 0 | 2 | 4 | 0 | 6 |
| *Poecilochroa variana* (C. L. Koch, 1839) | 0 | 0 | 1 | 0 | 1 |
| *Robertus arundineti* (O. P.-Cambridge, 1871) | 0 | 1 | 0 | 1 | 2 |
| *Robertus lividus* (Blackwall, 1836) | 0 | 0 | 1 | 0 | 1 |
| *Salticus zebraneus* (C. L. Koch, 1837) | 0 | 0 | 1 | 0 | 1 |
| *Sibianor aurocinctus* (Ohlert, 1865) | 0 | 0 | 1 | 0 | 1 |
| *Synageles subcingulatus* (Simon, 1878) | 0 | 0 | 1 | 0 | 1 |
| *Synageles venator* (Lucas, 1836) | 1 | 1 | 1 | 0 | 3 |
| *Synema globosum* (Fabricius, 1775) | 1 | 2 | 0 | 4 | 7 |
| *Talavera aequipes* (O. P.-Cambridge, 1871) | 0 | 1 | 0 | 0 | 1 |
| *Talavera aperta* (Miller, 1971) | 0 | 2 | 8 | 1 | 11 |
| *Talavera parvistyla* Logunov & Kronestedt, 2003 | 0 | 0 | 0 | 1 | 1 |
| *Tapinocyboides pygmaeus* (Menge, 1869) | 0 | 1 | 0 | 1 | 2 |
| *Tenuiphantes flavipes* (Blackwall, 1854) | 0 | 23 | 12 | 19 | 54 |
| *Tenuiphantes mengei* (Kulczyński, 1887) | 1 | 37 | 38 | 35 | 111 |
| *Tenuiphantes tenebricola* (Wider, 1834) | 0 | 0 | 1 | 0 | 1 |
| *Tenuiphantes tenuis* (Blackwall, 1852) | 0 | 3 | 2 | 1 | 6 |
| *Tetragnatha pinicola* L. Koch, 1870 | 0 | 0 | 2 | 1 | 3 |
| *Thanatus arenarius* L. Koch, 1872 | 0 | 1 | 0 | 0 | 1 |
| *Thomisus onustus* Walckenaer, 1805 | 0 | 1 | 0 | 0 | 1 |
| *Tiso vagans* (Blackwall, 1834) | 0 | 0 | 0 | 1 | 1 |
| *Titanoeca quadriguttata* (Hahn, 1833) | 0 | 1 | 0 | 0 | 1 |
| *Trichoncus affinis* Kulczyński, 1894 | 0 | 2 | 8 | 0 | 10 |
| *Trichopterna cito* (O. P.-Cambridge, 1872) | 67* | 9 | 0 | 1 | 77 |
| *Trochosa terricola* Thorell, 1856 | 0 | 1 | 0 | 1 | 2 |
| *Walckenaeria antica* (Wider, 1834) | 0 | 2 | 0 | 0 | 2 |
| *Xerolycosa miniata* (C. L. Koch, 1834) | 0 | 1 | 0 | 0 | 1 |
| *Xysticus bifasciatus* C. L. Koch, 1837 | 1 | 1 | 0 | 1 | 3 |
| *Xysticus cristatus* (Clerck, 1757) | 0 | 3 | 2 | 1 | 6 |
| *Xysticus erraticus* (Blackwall, 1834) | 1 | 0 | 0 | 0 | 1 |
| *Xysticus kochi* Thorell, 1872 | 1 | 1 | 0 | 0 | 2 |
| *Xysticus lanio* C. L. Koch, 1835 | 0 | 3 | 0 | 0 | 3 |
| *Zelotes apricorum* (L. Koch, 1876) | 0 | 1 | 0 | 0 | 1 |
| *Zelotes electus* (C. L. Koch, 1839) | 0 | 0 | 0 | 1 | 1 |
| *Zelotes exiguus* (Müller & Schenkel, 1895) | 0 | 1 | 2 | 0 | 3 |
| *Zodarion germanicum* (C. L. Koch, 1837) | 0 | 2 | 0 | 0 | 2 |
| *Zora silvestris* Kulczyński, 1897 | 0 | 0 | 18* | 1 | 19 |
| *Zora spinimana* (Sundevall, 1833) | 0 | 1 | 10* | 0 | 11 |
| Total species | 44 | 83 | 77 | 64 | 140 |
| Total individuals | 408 | 347 | 408 | 298 | 1461 |

Reference

Dufrêne M, Legendre P (1997) Species assemblages and indicator species: the need for a ﬂexible asymmetrical approach. *Ecol Monographs* 67: 345–366.
